# Attitudes toward e-learning of undergraduate students during COVID-19: Dataset from Indonesia

**DOI:** 10.1016/j.dib.2023.109380

**Published:** 2023-07-07

**Authors:** Irwanto Irwanto

**Affiliations:** Department of Chemistry Education, Universitas Negeri Jakarta, Jakarta 13220, Indonesia

**Keywords:** Attitudes toward e-learning, Distance learning, COVID-19, Undergraduate students, Indonesia

## Abstract

This article describes data on students’ attitudes toward e-learning at an Indonesian higher education institution during the pandemic period. This cross-sectional study was conducted with a total of 342 full-time students who studied at Universitas Negeri Jakarta in the 2022–2023 academic year. All respondents were determined using the convenience non-probability sampling method. To gather the data, the Attitude Scale Toward E-Learning (ASTEL) developed by Haznedar and Baran [1] was employed. The online survey was distributed to acquire the desired data on individual demographic characteristics (6 items), avoidance of e-learning (10 items), and tendency to e-learning (10 items). It was conducted from February to March 2023 with the support of lecturers. The dataset is available in the form of Microsoft Excel. The collected data provide new insights concerning students’ e-learning attitudes with regard to gender, age, grade level, daily duration of internet use, owner of a personal computer, and level of fear of contracting COVID-19. The dataset is made widely accessible to enable more critical and comprehensive investigations. The dataset will provide guidance to lecturers and policymakers in planning the effective use of e-learning and designing appropriate educational programs to enhance students’ achievement in technology-supported learning contexts.

**Specifications Table**

**Table:** 

Subject	Social Sciences
Specific subject area	Higher Education Educational Technology
Type of data	Tables
How the data were acquired	Data were collected via an online survey questionnaire (Google Form) at the beginning of the second semester of the 2022-2023 academic year. The Attitude Scale toward E-Learning (ASTEL) designed by Haznedar and Baran [1] was used to reveal students’ e-learning attitudes. The respondents were notified that they were invited to participate voluntarily and all information obtained would be kept confidential. They were required to answer all survey items.
Data format	Raw Analyzed
Description of data collection	The data were quantitative in nature. Using a convenience sampling method, a total of 342 valid responses were received and analyzed using IBM SPSS 25. Data were gathered using an online questionnaire which was distributed via the link sent by lecturers via WhatsApp groups between February and March 2023. The respondents were undergraduate students aged between 18–24 years (19.23±1.18) at Universitas Negeri Jakarta located in Jakarta with internet access. They used online learning platforms during this semester. The questionnaire comprised 20 items with a five-point Likert scale.
Data source location	Institution: Universitas Negeri Jakarta (UNJ) City/Town/Region: Jakarta Country: Indonesia Latitude: 6°10′30.00″ S, Longitude: 106°49′35.76″ E
Data accessibility	Repository name: Mendeley Data Data identification number: 10.17632/p4k894t6xc.1 Direct URL to data: https://data.mendeley.com/datasets/p4k894t6xc/1

## Value of the Data

• The dataset provides a significant contribution to capturing information about undergraduate students’ attitudes toward e-learning in Indonesia during the COVID-19 emergency.
• This dataset can be adapted for use in order to evaluate e-learning attitudes in primary and secondary education.
• The dataset will be valuable for educational researchers to compare students’ attitudes toward e-learning amidst the pandemic crisis with the new normal situation.
• This dataset will be useful to explore the effect of socio-demographic variables—i.e. gender, age, grade level, daily duration of internet use, owner of a personal computer, and level of fear of contracting COVID-19—on students’ attitudes toward e-learning.
• The dataset can serve as a good reference for policymakers, administrators, and researchers to gain valuable insights about e-learning attitudes as well as for educators to improve teaching and learning practices in higher education.
• This dataset can be replicated for comparative purposes; thus, further researchers can compare it with other datasets collected from different developing and developed countries, other higher education institutions, or similar works investigating e-learning attitudes in other areas.

## 1 Objective

The outbreak of the COVID-19 pandemic has driven the transition from traditional face-to-face courses to remote teaching settings. This situation has forced students to take courses with e-learning and spend more time with digital devices (e.g., smartphones, tablets, computers). With the increasing use of e-learning technologies throughout the coronavirus epidemic and given that distance education has been fully implemented in Indonesia, it is important to explore students’ positive or negative attitudes toward e-learning usage in terms of socio-demographic characteristics. This is due to the fact that students’ attitudes toward online learning are seen as a fundamental factor in achieving targeted learning outcomes [2]. In this regard, investigating students’ e-learning attitudes amidst the pandemic times will be decisive in evaluating the remote teaching process. This dataset [3] aims to provide useful information on measuring attitudes toward e-learning during the COVID-19 crisis. The dataset will be valuable for researchers who want to conduct further studies on attitudes toward online education in the context of a developing country, such as Indonesia. In addition, the accessible dataset is essential for educators and stakeholders to have a thorough understanding of students’ e-learning attitudes, so that more educational initiatives could be applied to accelerate the learning process.

## 2 Data Description

This study presents the dataset of a questionnaire on students’ attitudes toward e-learning usage during the COVID-19 outbreak. Data were collected through an online survey to ease the data collection and tabulation process. Data collection took about 5 weeks to complete (between February and March 2023). The demographic characteristics of the respondents sampled at a public university in Jakarta, Indonesia, are given in Table 1. Information about the descriptive characteristics of the respondents was coded as follows: gender (1=female, 2=male), age group (1 for 18–20 years, 2 for 21 years or more), grade level (1=first-year, 2=second-year, 3=third-year, 4=fourth-year), daily internet usage time (1 for 1–2 h, 2 for 3–4 h, 3 for 5–6 h, 4 for 7 h or more), having a personal computer (1=yes, 2=no), and level of fear of contracting COVID-19 (1=very fearful, 2=fearful slightly, 3=fearful, 4=not fearful at all).

**Table 1:** Descriptive characteristics of the respondents (*n*=342).

Variable	Categories	Code	Frequency	Percentage
Gender	Female	1	255	74.56
	Male	2	87	25.44
Age	18–20 years	1	290	84.80
	21 years or more	2	52	15.20
Grade level	First-year	1	162	47.37
	Second-year	2	81	23.68
	Third-year	3	66	19.30
	Fourth-year	4	33	9.65
Daily duration of Internet use	1–2 h	1	2	0.58
	3–4 h	2	35	10.23
	5–6 h	3	98	28.65
	More than 7 h	4	207	60.53
Owner of a personal computer	Yes	1	317	92.69
	No	2	25	7.31
Level of fear of contracting COVID-19	Very fearful	1	53	15.50
	Fearful	2	137	40.06
	Slightly fearful	3	119	34.80
	Not fearful at all	4	33	9.65

Of the 342 undergraduate students, 255 (74.56%) were females and 87 (25.44%) were males. It reflects that the number of females is thrice as high as male students. The gender ratio in the targeted sample was similar to that of the entire population. The majority of respondents were first-year students (47.37%), followed by second-year (23.68%), third-year (19.30%), and fourth-year students (9.65%). Age distribution included between 18–20 years (84.80 %) and 21 years or more (15.20 %). The mean age among the respondents was 19.23 years with a standard deviation of 1.18 years. This indicates that most of the respondents were young adults who could independently provide informed responses. More than half of the respondents (60.53%) spent 7 hours or more a day online, 28.65% spent 5–6 hours, 10.23% spent 3–4 hours, and only 0.58% spent 1–2 hours on the Internet. In addition, 317 (92.69%) had a computer, but 25 (7.31%) did not, which is in line with the findings of Çevik and Bakioğlu [2]. With respect to how much the respondents were afraid of the COVID-19 pandemic, 53 (15.50%) students were very fearful, 137 (40.06%) were fearful, 119 (34.80%) were slightly fearful, and 33 (9.65%) were not fearful at all. It is noteworthy that all respondents were full-time students and they came from urban areas.

This dataset contains two sub-dimensions; avoidance of e-learning and tendency to e-learning as described in Table 2. Tendency to e-learning (TE) was measured with 10 positive statements (TE1–TE10) and explained approach attitudes toward distance education (e.g., “*E-learning makes learning easier*”). Avoidance of e-learning (AE) was measured with 10 negative statements (AE1–AE10) and described avoidance attitudes toward distance education during the pandemic (e.g., “*The lack of face-to-face interaction in e-learning bothers me*”). Prior to data analysis, the 10 negative statements under the avoidance of e-learning subscale were reverse scored. Table 2 also displays Cronbach’s alpha (α) values of the two dimensions of e-learning attitudes. Cronbach’s alpha reliability coefficient for all sub-dimensions was found to be 0.90 in the current study and 0.93 by Haznedar and Baran [1]. According to Taber [4], the reliability coefficient of Cronbach’s alpha greater than 0.70 indicates relatively high internal consistency. Thus, no further changes were required, and the adopted instrument was used in data collection. The items on the questionnaire were nominal, ordinal, and scale. The raw data are accessible via https://data.mendeley.com/datasets/p4k894t6xc/1.

**Table 2:** The sub-dimensions, codes, number of items, and Cronbach’s alpha values of the survey.

Sub-dimension	Code	N of Items	Cronbach’s α
Avoidance of e-learning	AE	10	0.82
Tendency to e-learning	TE	10	0.91
All sub-dimensions		20	0.90

After receiving all the responses, the data were exported as an Excel file for importing to SPSS. To analyze the data, descriptive statistics including frequency, percentage, mean, standard deviation, variance, minimum and maximum, kurtosis, and skewness were calculated. Table 3 presents the results of the descriptive analysis of all items. The values of skewness and kurtosis were computed for the normality test. As explained by Hair et al. [5], the skewness and kurtosis values need to be in the range of –2 to +2 to be acceptable. As can be seen in Table 3, the data were normally distributed.

**Table 3:** Descriptive statistics of all items.

Item	Min	Max	M	SD	Variance	Skewness		Kurtosis	
						Statistic	Std. Error	Statistic	Std. Error
AE1	1	5	3.026	0.945	0.894	0.136	0.132	-0.069	0.263
AE2	1	5	3.044	0.993	0.986	-0.142	0.132	-0.323	0.263
AE3	1	5	3.015	1.032	1.064	-0.013	0.132	-0.585	0.263
AE4	1	5	3.234	0.927	0.860	-0.060	0.132	-0.308	0.263
AE5	1	5	2.415	1.130	1.276	0.556	0.132	-0.403	0.263
AE6	1	5	2.646	1.053	1.109	0.216	0.132	-0.463	0.263
AE7	1	5	3.175	0.962	0.925	-0.179	0.132	-0.079	0.263
AE8	1	5	4.029	0.866	0.750	-0.820	0.132	0.730	0.263
AE9	1	5	4.082	0.879	0.773	-0.837	0.132	0.480	0.263
AE10	1	5	2.810	1.177	1.386	0.244	0.132	-0.782	0.263
TE1	1	5	3.430	0.866	0.750	0.109	0.132	-0.128	0.263
TE2	1	5	2.994	0.825	0.680	0.137	0.132	0.176	0.263
TE3	1	5	2.936	0.867	0.752	-0.011	0.132	0.258	0.263
TE4	1	5	2.898	0.983	0.966	0.095	0.132	-0.223	0.263
TE5	1	5	2.813	0.929	0.862	0.182	0.132	0.075	0.263
TE6	1	5	3.167	0.882	0.779	0.157	0.132	0.410	0.263
TE7	1	5	2.980	0.911	0.829	0.158	0.132	0.138	0.263
TE8	1	5	3.026	1.008	1.017	-0.036	0.132	-0.328	0.263
TE9	1	5	3.471	0.971	0.942	-0.304	0.132	-0.125	0.263
TE10	1	5	3.383	0.819	0.671	-0.167	0.132	0.141	0.263

Aiming to evaluate students’ attitudes, the frequency distributions of the data are summarized in Table 4. It is clearly observed that the rating distributions for each item were concentrated in the “Neutral” and “Agree” options. This reflects that students’ attitudes toward e-learning tended to be positive. A possible reason for this result may be due to problems related to students’ fear of exposure to COVID-19. As a result, most students think that e-learning is necessary during a global pandemic. During distance learning, they can take courses from the comfort of their own homes and access learning materials at a time that is convenient for them [6]. Another reason why students have positive attitudes may be related to the advantages of e-learning such as time flexibility, self-paced, cost-effectiveness, and time-saving, as well as encouraging collaboration which has the potential to promote e-learning attitudes.

**Table 4:** Student responses regarding e-learning on each item.

Item	Strongly Disagree		Disagree		Neutral		Agree		Strongly Agree	
	F	%	F	%	F	%	F	%	F	%
AE1	16	4.678	75	21.930	160	46.784	66	19.298	25	7.310
AE2	24	7.018	68	19.883	140	40.936	89	26.023	21	6.140
AE3	23	6.725	86	25.146	120	35.088	89	26.023	24	7.018
AE4	9	2.632	61	17.836	141	41.228	103	30.117	28	8.187
AE5	79	23.099	120	35.088	84	24.561	40	11.696	19	5.556
AE6	51	14.912	102	29.825	122	35.673	51	14.912	16	4.678
AE7	18	5.263	52	15.205	151	44.152	94	27.485	27	7.895
AE8	4	1.170	12	3.509	63	18.421	154	45.029	109	31.871
AE9	3	0.877	14	4.094	59	17.251	142	41.520	124	36.257
AE10	46	13.450	103	30.117	97	28.363	62	18.129	34	9.942
TE1	4	1.170	31	9.064	164	47.953	100	29.240	43	12.573
TE2	9	2.632	77	22.515	176	51.462	67	19.591	13	3.801
TE3	18	5.263	73	21.345	177	51.754	61	17.836	13	3.801
TE4	26	7.602	86	25.146	147	42.982	63	18.421	20	5.848
TE5	25	7.310	94	27.485	159	46.491	48	14.035	16	4.678
TE6	11	3.216	46	13.450	190	55.556	65	19.006	30	8.772
TE7	16	4.678	76	22.222	170	49.708	59	17.251	21	6.140
TE8	24	7.018	72	21.053	142	41.520	79	23.099	25	7.310
TE9	11	3.216	35	10.234	128	37.427	118	34.503	50	14.620
TE10	5	1.462	34	9.942	153	44.737	125	36.550	25	7.310

## 3 Experimental Design, Materials, and Methods

This dataset primarily focused on students’ attitudes toward e-learning amidst the COVID-19 lockdown in Indonesia. In this quantitative study, the researcher used the cross-sectional method. The cross-sectional data were gathered during the global COVID-19 outbreak. A research ethics clearance and permission to collect data were requested from the Institutional Ethics Committee. It aims to ensure that the data collection protocol complies with safety requirements for all respondents. After being approved, the researcher then set a schedule to visit different lecturers to convey the purpose of the survey. All the recommendations provided by the institutional review board were also fully implemented to ensure that data collection was carried out properly adhering to all ethical considerations.

Considering data protection rules, only respondents who were at least 18 years old could participate in the study. They were selected through a non-probability convenience sampling technique to fill out the survey. This sampling method was chosen because it enabled the researcher to recruit respondents who were easily accessible [7]. In other words, the convenience approach allowed the researcher to target specific respondents with essential information regarding their online learning experiences amidst the lockdown due to increasing cases of COVID-19 to enrich the data. This approach was adopted in the majority of similar studies (e.g. [8]) to reach a wider range of students.

In the beginning, the original questionnaire [1] was translated into Indonesian from the original English version prior to data collection. Before being handed over to all respondents, the researcher carefully checked the Google Form first to see if there were any deficiencies, such as broken links, incomplete number of items and options, and unreadable questionnaires. To avoid duplicates in responses, settings in Google Forms were set to only record one response for each respondent. To reduce response bias, the researcher ensured that there was no pressure whatsoever that could affect responses and that there were no questions that would threaten the self-esteem of the respondents. Afterward, questionnaires were distributed as a survey to students during course hours with the help of lecturers. Students were asked to fill out an online questionnaire via the link provided. They answered the questionnaires voluntarily. The survey tool gathered data on six demographics of the respondents; gender, age, grade level, daily duration of internet use, owner of a personal computer, and level of fear of contracting COVID-19. Students were invited to take part in this research by completing a survey within four weeks. After these four weeks, the researcher reminded the respondents to complete the survey through their lecturers. Students were then given another week to do so. At the end of time, filling out the survey will be closed and the data will be downloaded for analysis.

The respondents were undergraduate students at UNJ, a public university located in the capital city of Indonesia. Before participating, all respondents were informed about the survey and provided informed consent including research objectives, risks, benefits, data confidentiality, respondent participation, name of contact researcher, and guarantee that the respondent can withdraw at any time. It is worth mentioning that the total number of students was 29,811. Of the total student population, 11,040 (37.03%) were males and 18,771 (62.97%) were females. This reflects that females constituted two-thirds of the student population. To accurately estimate the appropriate sample size for the dataset, Yamane’s [9] formula with a 95% confidence level was computed. Based on the calculations, the minimum targeted sample size was 395. At the end of the data-gathering process, there were a total of 452 clicks on the survey link. However, the response rate was lower than expected. Only 342 respondents completed the questionnaire with a response rate of 87%. To ensure anonymity, any information regarding respondents was removed after examining the distribution of their responses.

Data were collected from February 2 to March 8, 2023, using the ASTEL developed by Haznedar and Baran [1] and available in the Mendeley repository [3]. The ASTEL consisted of 20 items on a 5-point Likert scale with options “5-Strongly Agree”, “4-Agree”, “3-Neutral”, “2-Disagree” and “1-Strongly Disagree”. The questionnaire included two main sections; the first section included 6 demographic questions and the second section comprised 20 measurement items. Negative statements (Items AE1–AE10) were scored in reverse. All items in the questionnaire were obliged to be answered; thus, no missing data was found. The possible range of scores was 20–100. A high score in the ASTEL implies a high level of e-learning attitude. The questionnaire was self-administered by the students. The administration of questionnaires to respondents was based on their willingness to respond to the research instrument. They spent about 10–15 minutes completing the questionnaire. Upon completion, the submission of responses was carried out via Google Sheets. In the end, a total of 342 valid responses were received. All valid observations were accepted for further analysis. The data were then input into MS Excel and analyzed using SPSS software. The dataset was provided in the form of raw and analyzed data and made available in the Supplementary Materials.

The current research has a number of limitations. To carry out more rigorous and reliable research, it would be interesting to extend this work and increase sample size and response rates by motivating respondents to respond and providing incentives and rewards. In addition, to provide a more comprehensive picture of the factors influencing students’ preference for online distance learning, it is suggested to consider structural and institutional variables, such as self-efficacy, interest, academic motivation, institutional environment, infrastructures, and materials and resources that could explain the preferences of respondents. Lastly, future studies should adopt random sampling, expand the sampling area, and employ different triangulation methods to increase the generalizability of the findings. Although the data collected is limited (*n*=342), it can be used by researchers for further analysis to understand students’ e-learning attitudes during the COVID-19 epidemic in Indonesia. The data are very useful for improving higher education curricula so that appropriate interventions can be designed to accelerate the process of technology adoption in order to prepare graduates who are technologically literate for the future.

## Ethics Statements

The study was carried out following the procedures outlined by the Declaration of Helsinki and was approved by the Research Ethics Committee of Universitas Negeri Jakarta [Ref. No.: 897/UN39.14/PT/XII/2022]. It should be noted that all respondents were adults aged 18 and above. Prior to starting the survey, students were asked to carefully read the participant information letter linked on the front page of the questionnaire. It was intended that all respondents could make a decision about their consent to proceed with the survey. Respondents could withdraw at any time before completing the survey by simply closing the survey link in their browser. Once clicking the “Submit” button at the end of the questionnaire, they were deemed to have agreed to voluntarily participate in the study.

## CRediT authorship contribution statement

**Irwanto Irwanto:** Conceptualization, Methodology, Investigation, Data curation, Visualization, Supervision, Validation, Software, Writing – original draft, Writing – review & editing.

## Declaration of Competing Interest

The author declares to have no known competing financial interests or personal relationships which have, or could be perceived to have, influenced the work reported in this paper.

## Data Availability

Attitudes toward e-learning of undergraduate students during COVID-19: Dataset from Indonesia (Original data) (Mendeley Data).

## References

[1] Haznedar Ö., Baran B. (2012). Development of a general attitude scale towards e-learning for faculty of education students. Eğitim Teknol. Kuram ve Uygul..

[2] Çevik M., Bakioğlu B. (2022). Investigating students’ e-learning attitudes in times of crisis (COVID-19 pandemic). Educ. Inf. Technol..

[3] Irwanto I. (2023). Attitudes toward e-learning of undergraduate students during COVID-19: dataset from Indonesia. Mendeley Data.

[4] Taber K.S. (2018). The use of Cronbach's alpha when developing and reporting research instruments in science education. Res. Sci. Educ..

[5] Hair J.F., Hult G.T.M., Ringle C.M., Sarstedt M. (2022).

[6] Ninsiana W., Gabidullina F.I., Widodo M., Patra I., Pallathadka H., Alkhateeb D.A.A.M., Zainal A.G., Gheisari A. (2022). High school students’ attitudes towards e-learning and impacts of online instruction on their general English learning: challenges and issues. Educ. Res. Int..

[7] Fraenkel J.R., Wallen N.E., Hyun H.H. (2012).

[8] Aristovnik A., Keržič D., Ravšelj D., Tomaževič N., Umek L. (2021). Impacts of the Covid-19 pandemic on life of higher education students: global survey dataset from the first wave. Data Br..

[9] Yamane T. (1967).

